# Transferring interprofessional education concepts across sites – experiences and recommendations for practice

**DOI:** 10.3205/zma001529

**Published:** 2022-02-15

**Authors:** Eva Bibrack, Henrike Horneff, Katja Krumm, Jutta Hinrichs, Mira Mette

**Affiliations:** 1 Technische Universität Dresden, Carl Gustav Carus Faculty of Medicine, Inter-Professional Medical Training Center (MITZ), Dresden, Germany; 2 University Leipzig, Medical Faculty, LernKlinik Leipzig, Skills- and Simulation Centre, Leipzig, Germany; 3 Academy of the University Hospital Mannheim GmbH, School of Physiotherapy, Mannheim, Germany; 4 Medical Faculty Mannheim, Heidelberg University, Division of Study and Teaching Development, Mannheim, Germany

**Keywords:** interprofessional education, transfer, curriculum development, interprofessional relations

## Abstract

**Aim: **Interprofessional education for health care professionals should be anchored at all training and study locations across Germany. In cooperation with the Medical Faculty Mannheim, an education concept trialed there, namely a longitudinal interprofessional learning sequence, was transferred and adapted to the Medical Faculty Dresden as part of the “Operation Team” support program. Here, the structured analysis and evaluation of the knowledge transfer experience is presented from the perspective of the transferee. From these findings, recommendations are derived for the planning of knowledge transfer projects.

**Methods:** The consulting work between the two faculties was listed chronologically including knowledge transfer outcomes and was described and analyzed using the comparative categories identified in sociological systems theory and in the knowledge transfer literature. In addition, knowledge transfer outcomes were categorized according to their use and their relevance to the progress of the project was assessed.

**Results: **The coordination teams initiated 13 consulting sessions, primarily held virtually or by telephone. From these, 36 knowledge transfer outcomes were identified, of which most were of high relevance for the transferee in all use categories. The knowledge transfer core themes were of a strategic (e.g. the consolidation of interprofessional teaching) and content-based/didactic-methodological nature (e.g. interprofessional session design, tutor training).

**Conclusion:** The consulting sessions played a major role in facilitating the establishment of two interprofessional learning sequences and the piloting of the associated sessions at the Dresden site. The recommendations derived for a successful transfer could also be of help for other transfer projects.

## Zusammenfassung

**Zielsetzung: **Interprofessionelle Lehrangebote sollen deutschlandweit an allen Ausbildungs- und Studienorten für Gesundheitsprofessionen verankert werden. Im Rahmen der Förderlinie „Operation Team“ wurde in Kooperation mit der Medizinischen Fakultät Mannheim das dort erprobte Lehrkonzept eines longitudinalen interprofessionellen Lernstrangs an die Medizinische Fakultät Dresden übertragen und angepasst. Die strukturierte Analyse und Bewertung der Erfahrungen des Wissenstransfers wird aus Sicht des Transfernehmenden dargestellt. Daraus werden Empfehlungen für die Planung von Wissenstransferprojekten abgeleitet.

**Methodik: **Die Beratungssituationen zwischen beiden Fakultäten wurden mit Wissenstransferoutcomes chronologisch aufgelistet und mithilfe der in der soziologischen Systemtheorie und Literatur zu Wissenstransfer identifizierten Vergleichskategorien beschrieben und analysiert. Die Wissenstransferoutcomes wurden zusätzlich nach ihrer Verwertung kategorisiert und ihre Relevanz für den Projektfortschritt eingeschätzt.

**Ergebnisse: **Es fanden 13 Beratungen hauptsächlich in Form elektronischer Kommunikation auf Initiative der Koordinationsteams statt. Daraus wurden 36 Wissenstransferoutcomes mit größtenteils hoher Relevanz für den Transfernehmenden in allen Verwertungskategorien identifiziert. Grundthemen des Wissenstransfers waren strategischer (z.B. Verstetigung interprofessioneller Lehre) und inhaltlich/didaktisch-methodischer Natur (z.B. Konzeption interprofessioneller Lehre, Schulung von Lernbegleitenden).

**Schlussfolgerung: **Durch die Beratungen wurde der Aufbau zweier interprofessioneller Lernstränge und die Pilotierung der dazugehörigen Lehrveranstaltungen am Standort Dresden maßgeblich erleichtert. Für andere Transferprojekte können die abgeleiteten Empfehlungen für einen gelingenden Transfer hilfreich sein.

## Introduction

Over the last decade, there have been increasing calls in Germany for the nationwide introduction of interprofessional (IP) education [1], [2], [3] in order to better prepare future health care professionals for collaboration with other professional groups. Accordingly, a range of pilot projects, each with site-specific IP education concepts, have emerged, in particular as part of the “Operation Team” [4], [5], [6] support program. As a result, the implementation and curricular integration of IP sessions in health care profession training/study programs has been largely heterogenous [7]. There is still a need for development, as the current draft of the new German medical licensure act [8], the revised National Competence-based Learning Objectives Catalogue for Medicine [http://www.nklm.de], and the Topic Catalogue for Medicine [9] are all aiming to introduce targeted and mandatory IP sessions across all medical faculties in Germany. Inter alia, the position paper of the GMA committee “Interprofessionelle Ausbildung in den Gesundheitsberufen” [Interprofessional Education in the Health Care Professions] recommends the creation of long-term organizational structures and the efficient and comprehensive use of resources in the implementation of IP education [10]. These recommendations were taken up in the third support phase of the Robert Bosch Stiftung’s “Operation Team” program [11], [12]. This was achieved through the targeted promotion of the transfer of IP education concepts which had already been trialed, and positively evaluated under the specific conditions of the primary sites [13] to additional sites. The aim here was for the sites to share their knowledge and findings on already established IP education formats, thus increasing the impact. As numerous barriers must usually be overcome in the development and implementation of IP education [9], [11], [14], [15], a transfer is an opportunity to communicate both the positive and negative experiences and impacts in the introduction and establishment of IP sessions, thus rendering them usable for other sites. It also serves to promote active networking between the various stakeholders at play. 

Systematic project transfer is widely regarded as a proven method of multiplying ideas, concepts, and approaches, while avoiding fundamentally rethinking and resolving problems with similar objectives [16]. Successful projects are reviewed with regard to their potential for transfer, before being modified in line with the specificities of the new site and implemented in conversation with the project partner. Knowledge transfer is well suited to sharing project knowledge and experience with others via a range of transfer methods, including disseminating knowledge and experience via open source, training, consulting sessions, or certification [16]. A number of options present themselves in the design of project transfers: Projects can be transferred in their entirety with all their complexities, or partially in the form of individual project elements [16]. Similar to consulting, knowledge transfer means expertise, knowledge, or skills that are passed from one party (the transferor) to a second party (the transferee) in order to provide support or solve problems [17].

To date, there is no evidence on the potential of the inter-site transfer of IP education concepts to succeed. Accordingly, this report focuses on a structured analysis and evaluation of the knowledge transfer experience from the perspective of the transferee. From this, recommendations for the planning of knowledge transfer projects will be derived. 

## Project description

The aim at the Dresden site was to integrate longitudinal, topic-specific learning sequences, each with at least three sessions, into compulsory teaching as part of the “Carus Interprofessional” project. It was planned that at least two professions would jointly participate in these IP learning sequences, for instance medical students with trainees from either physiotherapy, midwifery, or nursing. 

To support a transfer of IP education concepts [12], it was possible to utilize projects from previous funding phases [5], [6]. The education concept was selected for the transfer on the basis of a number of criteria which had been deemed by participating stakeholders from the Medical Faculty Dresden (the transferee) to play a decisive role in the successful transfer of IP sessions as well as in their hopefully permanent implementation. The key criteria behind the decision were:

1. The sustainability of the IP education concept
2. Its transferability to other educational programs
3. Its structural adaptability
4. Its innovation/design potential

**Figure 1:**
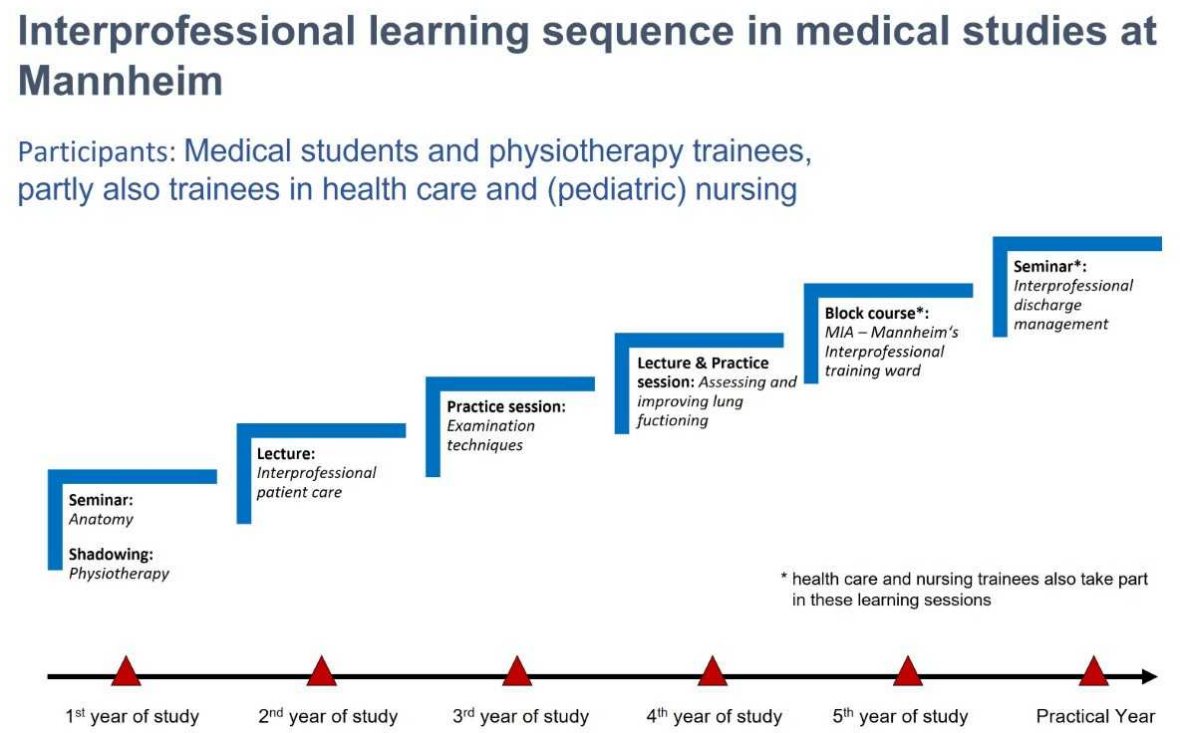
Mannheim’s interprofessional learning sequence Note: Cooperation partner: School of Physiotherapy, Academy of University Hospital Mannheim GmbH.

The Medical Faculty Mannheim was identified as a suitable partner for the cooperation and transfer provider in light of it having an education concept involving forming a longitudinal IP learning sequence with different IP sessions (see figure 1 (Fig. 1)) [18], [19], [20]. 

**Figure 2:**
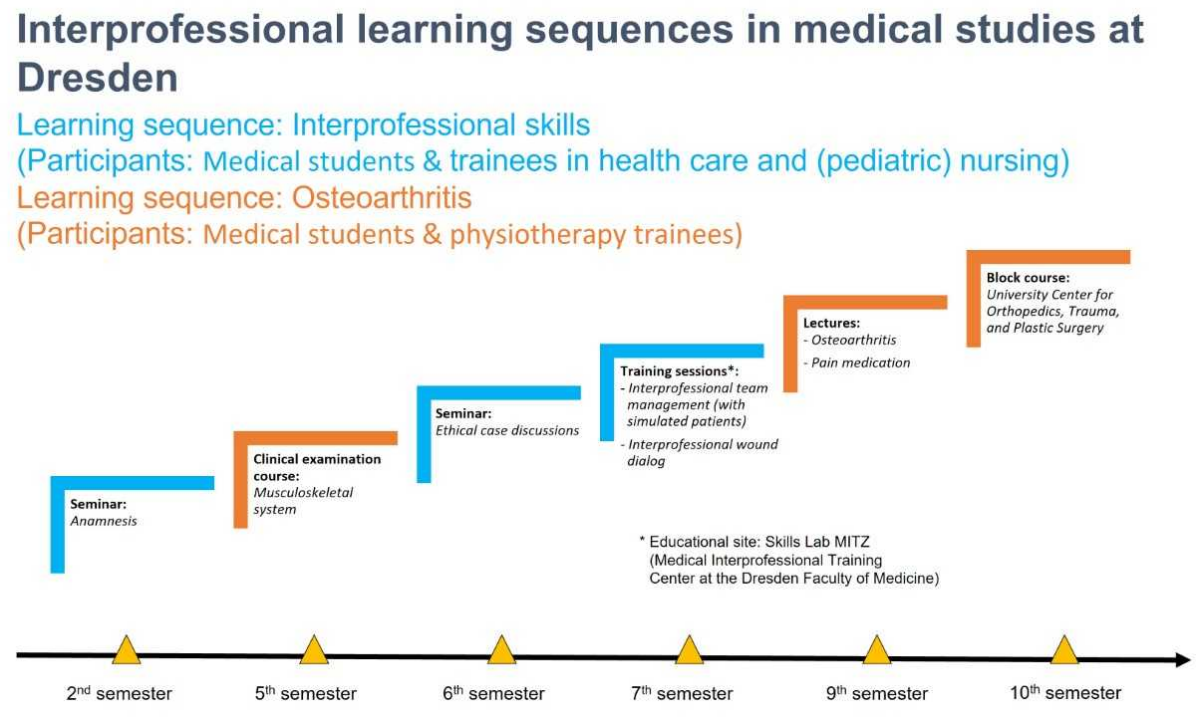
Dresden’s interprofessional learning sequences “Carus Interprofessional”. Notes: Cooperation partner for “Osteoarthritis”: Carus Physiotherapy Training Area & the University Center for Orthopedics, Trauma, and Plastic Surgery OUPC at the University Medical Center Dresden; Cooperation partner for “Interprofessional skills”: Carus Academy Nursing Training Area & General Medicine/Ethics, History, and Theory of Medicine at the University Medical Center Dresden.

The similar approach between the two faculties suggested great potential for transfer to Dresden through the experiences of the Mannheim Faculty in the development, trialing, evaluation, and curricular anchoring of an IP learning sequence. Over the course of the project (October 1, 2018 – September 30, 2021), the Mannheim education concept was adapted for the Dresden context. Accordingly, several IP sessions were developed in learning sequences focusing on two main topics (see figure 2 (Fig. 2)). 

## Methods

The knowledge transfer is defined as a communicative process [21]. In the knowledge transfer process, the interactions between the project partners were compiled chronologically in an overview as consulting sessions. E-mail exchanges, the minutes of telephone/video conference calls, and the documents provided were used as the basis for this, from which specific knowledge transfer outcomes were derived for the transferees. Relevant aspects for the transfer were identified in the literature on sociological systems theory [22] and knowledge transfer [23], [24]. These criteria were used to classify each consulting session and all knowledge transfer outcomes [22], [23], [24]. A predominantly qualitative approach was taken to the evaluation of the transfer process. The two transfer partners jointly compiled the overview as well as the description of the consulting sessions. The transferee extracted the specific transfer outcomes from the consulting sessions and assessed their relevance. The relevance of an outcome was rated according to the extent to which the knowledge gained from the transfer impacted the project’s progress. The evaluation criteria are shown and explained in table 1 (Tab. 1). 

The transferee was primarily responsible for recapitulating and evaluating how the transfer outcomes were specifically used in the implementation of IP learning sequence. The transfer project team discussed and consolidated differently rated elements. Core themes, that is to say thematic intersections, were identified by clustering the transfer outcomes. In addition, the use of the transfer results and the relevance for the project’s progress were used as the basis for the quantitative evaluation of the frequency of transfer activities and outcomes. 

## Results

Table 2 (Tab. 2) details the individuals involved in the transfer process. In the majority of cases, the knowledge transfer took place via telephone or e-mail. In light of the spatial distance and staffing changes, two face-to-face meetings took place over the course of the two-year consulting period (May 2018 – October 2020), including three consulting sessions during a visit of the transferee to the transferor and one consulting session at a symposium. Two virtual face-to-face consulting sessions took place in 2020 using web and video conferencing tools. The project established that the initiative of the IP education coordination teams at both sites was indispensable in knowledge transfer.

A total of 13 consulting sessions took place, ten of which in the project’s first year and three in its second. Consultations took place between the two project partners on an as-needed basis. Pre-defined criteria were used to compile and classify the individual consulting sessions in an overview (for examples, see table 3 (Tab. 3), complete overview in the attachment 1 ). 

The majority of the consulting sessions (nine) were initiated by the transferee (user pull), two by the transferor (producer push). Over the course of the process, two sessions were characterized by joint mutual input and the joint finding of suitable approaches or solutions (exchange). 

The consultations resulted in a total of 36 knowledge transfer outcomes (26 in the first year of the project, ten in the second), of which 69% were rated by the transferee as either moderately or highly relevant in achieving the aim of the project, that is to say in implementing IP learning sequences at the Dresden site (see table 4 (Tab. 4)). Irrelevant transfer outcomes (22%) primarily related to administrative matters (e.g. contract formalities). The use of the transfer outcomes was instrumental (33%), conceptual (42%), symbolic (8%), or had no direct use (17%) for the transferee.

The transfer outcomes that were highly relevant for the transferee in achieving their goals resulted from all the different forms of transfer used (face-to-face meetings, shadowing, e-mail exchanges, telephone exchanges, video conferencing). After clustering, the core themes of the outcomes were either strategic or content-based/didactic-methodological in nature. The strategic transfer outcomes included the core themes “consolidation of IP sessions” (e.g. study commission, financing, legal framework for course regulations) and “project planning” (preparation of project group meetings, frequency of working group meetings, administrative information for project implementation). The content-based outcomes related to the “IP session design” (course planning, material preparation, selection of suitable teaching methods), “tutor training for IP sessions”, and the “further development of IP sessions” (course evaluation and adaptation).

## Discussion

The following section involves a discussion of the results according to three aspects: the setting of the consulting session, the activities between transferor and transferee, and knowledge transfer activities and outcomes. 

### Consulting session setting 

Within the consulting process, the implementation of different forms of knowledge transfer enabled the generation of relevant knowledge transfer outcomes. To what extent increased face-to-face exchange would have positively impacted the emergence of outcomes is nonetheless open to discussion. The spatial distance between the two locations limited the feasibility of holding face-to-face consulting sessions, but video conferences could have taken place with greater frequency. With hindsight, both the transferor and transferee considered this form of knowledge transfer as highly beneficial, as it facilitates the creation of personal connections, which in turn significantly smooths exchange [24]. 

The points in time at which the 13 consulting sessions were implemented were chosen intuitively, largely by the transferee. However, the uneven distribution of the consulting sessions (ten in the first year, three in the second) does not point to a correlation between consultation quantity and consultation quality. The findings indicate that a comprehensive consulting appointment can result in a range of relevant transfer outcomes. Initially, the launch of the project necessitated the coordination of more frequent, smaller consulting sessions, as the transferee was unable to draw on any prior experience of the development and implementation of longitudinal IP learning sequences. 

It is possible that the use of a consulting plan based on an actual-target-gap analysis could have engendered a more decisive approach centered around a consulting concept. Furthermore, a kick-off meeting at the beginning of the project could have served to clearly set out the needs and expectations of the transferee, thus bringing greater focus and efficiency to the transfer. In addition, regular updates from the transferee regarding the status of the actual-target gap would have been of use. Such updates would have made it possible to modify the transfer process where necessary and for the transferor to prepare further consulting sessions accordingly. 

#### Activities between transferor and transferee 

The coordinators of IP education at the transferee’s institution involved people from all the different target groups, namely students and trainees as well as administrators and teaching supervisors. However, within the consulting process, exchange occurred almost exclusively between the coordination teams. 

Students and trainees were never directly involved in the consulting sessions, with staff managing students, administrators, student and trainee teaching staff only sporadically engaged in the process. Had the coordination team explicitly requested all target groups play a specific role in the transfer with defined consulting themes, this could have promoted cross-site interactions between actors other than the coordination team. Quite possibly, the project may have also uncovered further relevant outcomes, had the coordinators established greater contact between IP teaching staff across both locations. For instance, training and qualification in the form of a workshop between transferor and transferee teaching staff could well have supported the design and implementation of sessions in the osteoarthritis learning sequence. In turn, this could have established closer contact between the two transfer partners, from which the transferor could also have benefited. In coordinating IP education, it is vital to include all relevant actors [4].

#### Knowledge transfer activities and outcomes

The consulting process spanned a broad range of themes, with the consultation work serving as a key activity within the project coordination work. Without the IP education coordinators providing momentum for consultations within the transfer process, there would have been a lack of initiative to discuss and therefore help in the resolution of problems. 

Rated by the transferee as specific and practice-focused, and thus particularly fruitful, the overview of the consulting sessions (see attachment 1 ) highlights that the instrumental and concept-based transfer outcomes in particular play a central role in the consulting process. It is possible that more in-depth initial exchange between the respective managements or those responsible for teaching may also have generated more symbolic transfer outcomes. In turn, this may have helped eliminate doubts in the current project regarding the necessity of implementing the planned IP learning sequences in a more targeted manner. The varying structural and hierarchical natures of different educational institutions mean that symbolic transfer outcomes can be of crucial importance. This is because awareness of the importance and significance of IP education and the associated barriers [9], [11], [14], [15] may be less pronounced elsewhere, engendering a situation whereby necessary factors for the implementation of IP education must be repeatedly clarified and renegotiated. 

The quantitative findings (frequency of knowledge transfer activities) reveal that there were only two exchange-based consulting sessions where transferor and transferee collaborated in terms of joint input and joint decision making. This was possibly a result of the brevity of the consulting period, which in any case focused on utilizing the transferor’s experience. A longer consulting period may have generated more IP education experience at the transferee faculty, thereby enabling greater exchange regarding joint solutions, decisions, or activities. The spatial distance, multiple staff changes in the transferee’s project coordination team, and a needs-based consulting approach may also have played a role in keeping the focus on user pull. Having a constant team at both sites may have facilitated a more in-depth advice-sharing and therefore more exchange. Taken retrospectively, both transfer partners missed a number of opportunities for exchange in the form of cross-site collaboration. COVID-19 restrictions, for instance, could have laid the foundations for collaboration on the creation of IP e-learning or to intentionally promote exchange between IP teaching staff. It seems possible that consulting sessions based on exchange may have produced a greater number of highly relevant outcomes. In addition, such outcomes would not only have been key in advancing IP education at the transferee institution, but also at the transferor. Equally, they could have promoted closer cooperation between the two sites, including possibly on future projects. 

The present report, based on the authors’ experiences, describes, analyzes, and evaluates the consultation process from a single transfer project. The evaluation criteria employed, which were derived from the literature on knowledge transfer and sociological systems theory, have proven to be suitable. As such, this has facilitated the formulation of a series of general recommendations for the successful implementation of knowledge transfer projects. These recommendations include site- and project-specific framework conditions.

## Conclusion & recommendations

Knowledge transfer makes it possible to transfer tried and tested IP education concepts to other sites by drawing on the experience of the transferor. Overall, the IP education concept knowledge transfer from Mannheim to Dresden can be seen to have been a success. The transfer enabled several successful implementation strategies to be adopted and avoided potential barriers at the new location. Within the consulting process, the great degree of flexibility at spatial level and regarding the forms of communication facilitated rapid responses to changing circumstances. In addition, the willingness on the part of the transferor to share pre-existing project experiences freely and transparently point out opportunities and limitations was considered particularly enriching. Nonetheless, more structured preparation would have brought greater benefit still to the consulting and transfer process. On the basis of the literature employed and the specific project experiences, the following recommendations for the planning of knowledge transfer projects were derived, centering around the types of transfer activity (producer push, user pull, exchange):

- User pull (on the initiative of the transferee):
  - Set out specific transfer aims and expectations for the transferor;
  - Determine criteria for the selection of a suitable transferor and prioritize where necessary, e.g. project similarity, spatial distance, the need for information, forms of communication, expectations for the cooperation, and, if necessary, consulting costs;
  - Check project progress systematically.
- Producer push (on the initiative of the transferor):
  - Offer specific transfer help at both the content and implementation levels;
  - Communicate experiences both at specialist level but also with knowledge transfer projects;
  - Specify expectations and requirements for knowledge transfer, e.g. involvement of specific persons/functions/committees, specific timeframe;
  - Check project progress systematically.
- Exchange (joint initiative and cooperation of both transfer partners):
  - Develop transfer concept on the basis of a specific needs analysis and an actual-target-gap analysis;
  - Set out form and scope of cooperation (preferably face-to-face) incl. communications (reporting system);
  - Coordinate a project plan with milestones, regularly check the project status, and adapt the plan to the needs of the transferee;
  - Raise level of commitment through contractual regulations on both sides;
  - Finally, critically evaluate the transfer process, including perspectives for future longer-term cooperation on other projects, areas, or constellations.

These recommendations should facilitate the targeted design of the process of transferring knowledge to other locations. 

## Authorship

The authors Eva Bibrack and Henrike Horneff share the first authorship.

## Competing interests

The authors declare that they have no competing interests. 

## Supplementary Material

General overview analysis of the consulting sessions within the knowledge transfer process

## Figures and Tables

**Table 1:**
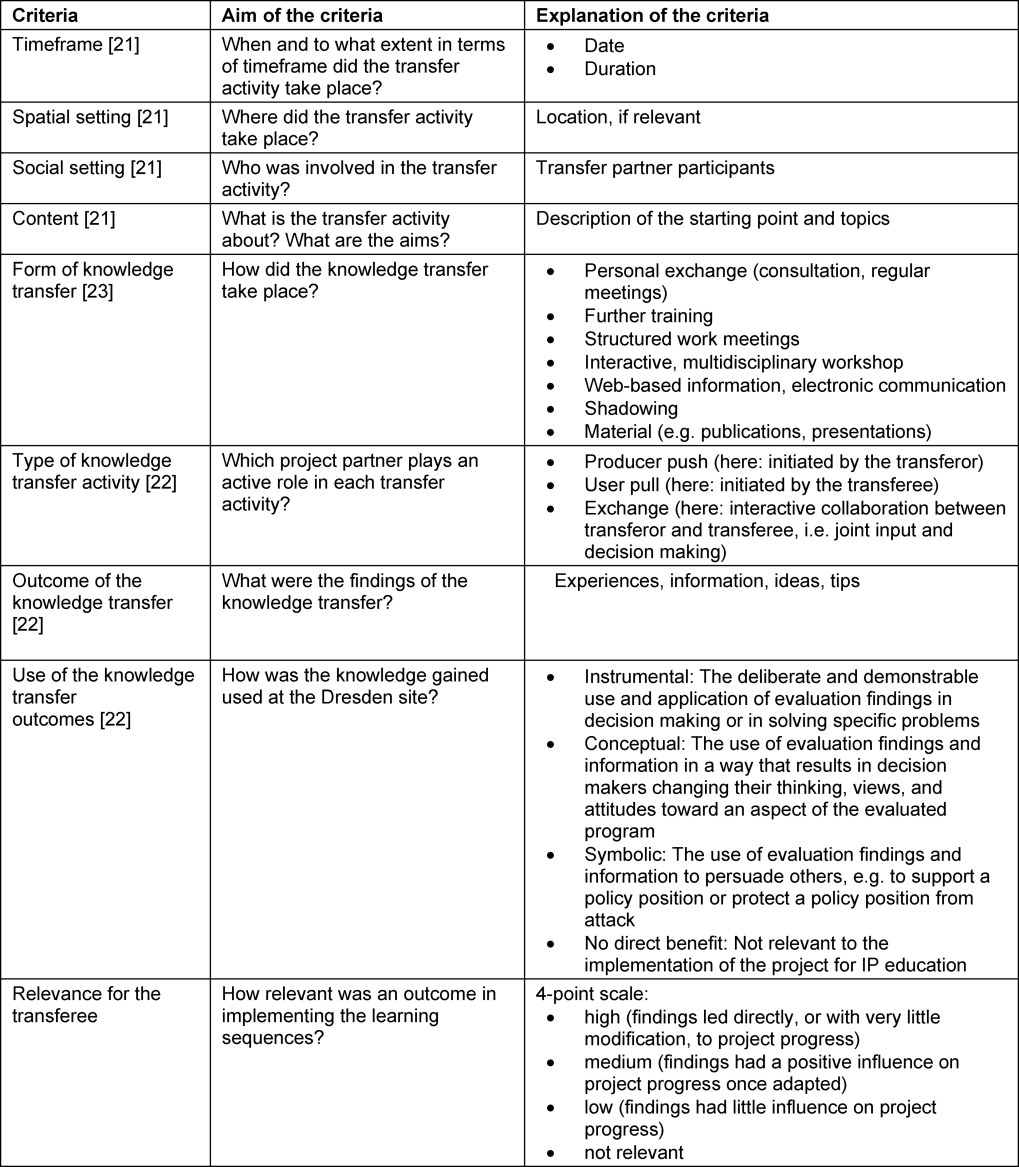
Evaluation criteria overview

**Table 2:**
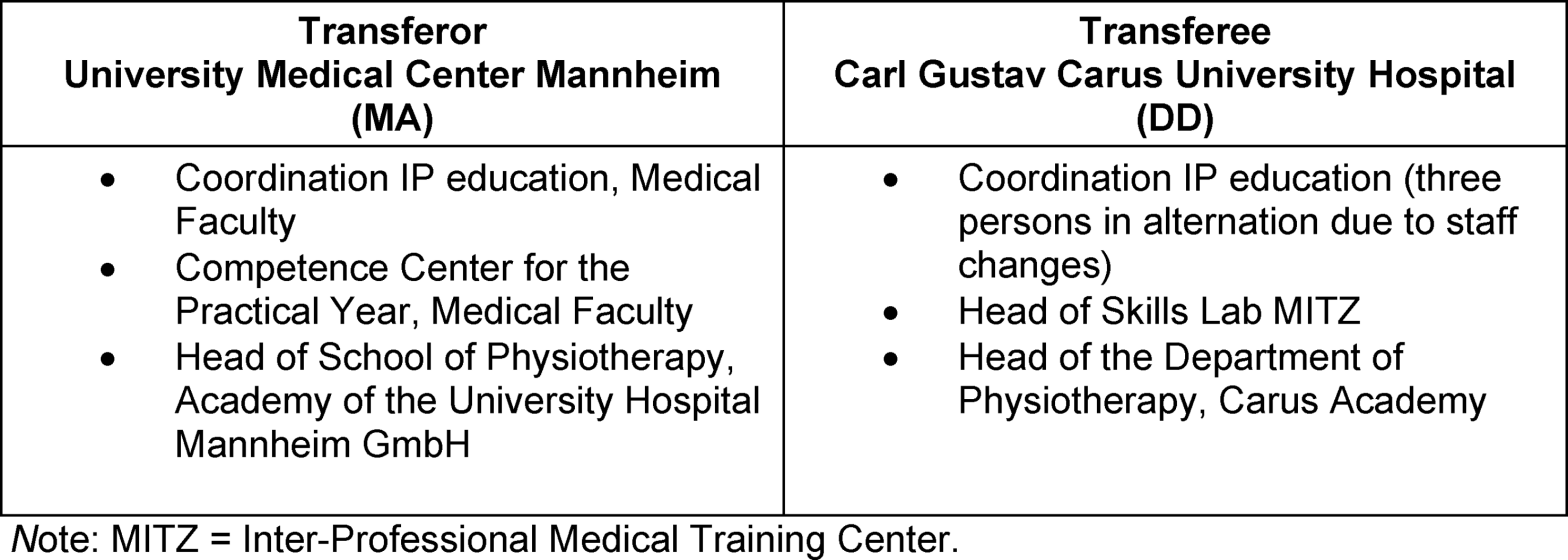
Primary participants in the IP education concept transfer process

**Table 3:**
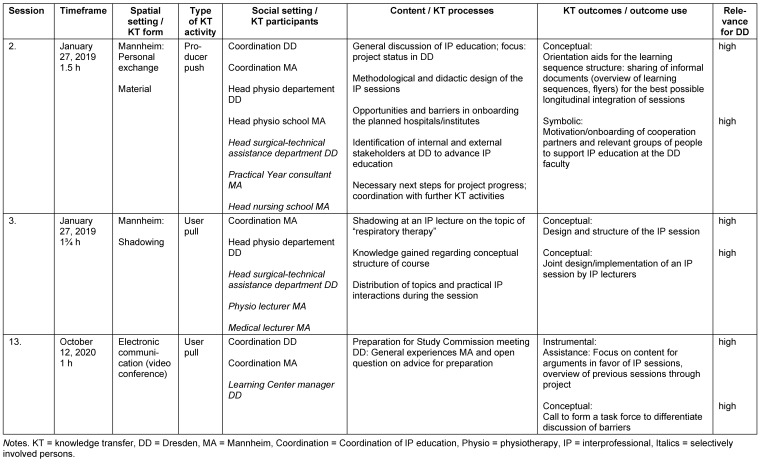
Excerpt from the overview of the categorized and classified consulting sessions

**Table 4:**
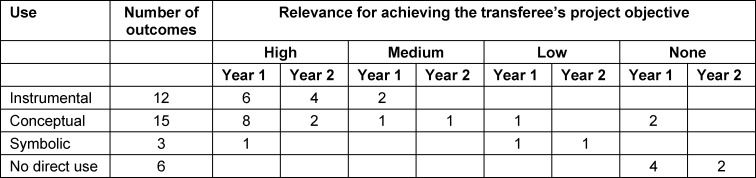
Overview of the knowledge transfer outcomes by use, relevance, and project year
