# Supplementary material for: Transferring interprofessional education concepts across sites – experiences and recommendations for practice
Source: GMS J Med Educ. 2022 Feb 15;39(1):Doc8. doi: 10.3205/zma001529 (PMC8953188; doi:10.3205/zma001529)
Supplement: General overview analysis of the consulting sessions within the knowledge transfer process [file JME-39-8-s-001.pdf]

Attachment 1: General overview analysis of the consulting sessions within the knowledge transfer process

Consulting process preparation							
**Session**	**Time-frame**	**Spatial setting/ form of communication**	**Type of activity**	**Social setting/ participants**	**Content/ KT processes**	**Outcomes/outcome use**	**Relevance for DD**
0.1	May 2018	Electronic communication (phone calls, e-mails)	User pull	Coordination MA<br><br>Coordination DD	Contacting the IP education projects funded by the Robert Bosch Stiftung after the marketplace event.<br><br>Request for and approval by transfer partners; confirmation letter prepared as an appendix for the funding application<br><br>Consultation and review of funding application at the Robert Bosch Stiftung	Conceptual:<br>Importance of support of key decision makers at the Medical Faculty DD, University Hospital DD, and Carus Academy → letters of support collected<br><br>Instrumental:<br>Sending a letter of endorsement for the grant application, confirming willingness to cooperate between DD and MA	high<br><br>high
0.2	November/ December 2018	Electronic communication (phone calls, e-mails)	Exchange	Coordination MA<br><br>Coordination DD	Contract and financing design for the consulting cooperation<br><br>Need for a service agreement to regulate the consulting work within the transfer process<br><br>Organizational arrangements for shadowing date in January	Ø:<br>Service agreement issued that specifically sets out the consulting work to be provided<br><br>Conceptual:<br>Shadowing in MA to garner direct experience of the IP sessions → better perception of the practical implementation of teaching formats	none<br><br>high

N.B.: DD = Dresden, MA = Mannheim, Coordination = Coordination IP education.

Knowledge transfer process							
**Session**	**Timeframe**	**Spatial setting/ KT form**	**Type of KT activity**	**Social setting/ KT participants**	**Content/ KT processes**	**KT outcomes/outcome use**	**Relevance for DD**
1.	January 27, 2019<br>4 hours	Mannheim: shadowing	Producer push	Coordination DD<br><br>Physio manager DD<br><br>*Surgical-technical assistance manager DD*<br><br>*Nursing supervisor MA*	Informative tour of the IP training ward, getting to know MA learning sequence on site	Symbolic:<br>Onboard motivated key individuals, consider structural specifics, internal and external importance of public relations project	low
2.	January 27, 2019<br>1.5 hours	Mannheim: Personal exchange<br><br>Material	Producer push	Coordination DD<br><br>Coordination MA<br><br>Head physio department DD<br><br>Head physio school MA<br><br>*Head surgical-technical assistance department DD*<br><br>*Practical Year consultant MA*<br><br>*Head nursing school MA*	General discussion of IP education; focus: project status in DD<br><br>Methodological and didactic design of the IP sessions<br><br>Opportunities and barriers in onboarding the planned hospitals/institutes<br><br>Identification of internal and external stakeholders at DD to advance IP education<br><br>Necessary next steps for project progress; coordination with further KT activities	Conceptual:<br>Orientation aids for the learning sequence structure: sharing of informal documents (overview of learning sequences, flyers) for the best possible longitudinal integration of sessions<br><br>Symbolic:<br>Motivation/onboarding of cooperation partners and relevant groups of people to support IP education at the DD faculty	high<br><br>high

Knowledge transfer process							
**Session**	**Timeframe**	**Spatial setting/ KT form**	**Type of KT activity**	**Social setting/ KT participants**	**Content/ KT processes**	**KT outcomes/outcome use**	**Relevance for DD**
3.	January 27, 2019<br>1¾ hours	Mannheim: shadowing	User pull	Coordination MA<br><br>Physio manager DD<br><br>*Surgical-technical assistance manager DD*<br><br>*Physio lecturer MA*<br><br>*Medical lecturer MA*	Shadowing at an IP lecture on the topic of “respiratory therapy”<br><br>Knowledge gained regarding conceptual structure of course<br><br>Distribution of topics and practical IP interactions during the session	Conceptual:<br>Design and structure of the IP session<br><br>Conceptual:<br>Joint design/implementation of an IP session by IP lecturers	high<br><br>high

Knowledge transfer process							
**Session**	**Timeframe**	**Spatial setting/ KT form**	**Type of KT activity**	**Social setting/ KT participants**	**Content/ KT processes**	**KT outcomes/outcome use**	**Relevance for DD**
4.	January 27, 2019<br>1.5 hours	Mannheim: shadowing	User pull	Coordination DD<br><br>Head physio department DD<br><br>Head physio school MA<br><br>*Physio instructor MA*<br><br>*Medical lecturer MA*<br><br>*Head surgical-technical assistance department DD*	Shadowing at IP practice session: “Assessing and improving lung function”<br><br>Demonstration of IP group participant distribution<br><br>Observation of IP small group work on subsessions as a method<br><br>Gaining experience of the different emphasis of the respective subsessions instructors (medical lecturers or physio teaching staff)<br><br>Experience of the direct IP exchange and the expansion and mutual support of participants<br><br>Experience of direct participant feedback on the session<br><br>Exchange with lecturers on IP session design and the positive impact and ‘barriers’ of a practical IP session	Conceptual:<br>Stronger understanding on the design and process of a practice session<br><br>Instrumental:<br>Participant feedback very valuable for the conceptual design of the planned session<br><br>Conceptual:<br>Ideas for the design and evaluation of a IP practice session	high<br><br>high<br><br>high

Knowledge transfer process							
**Session**	**Timeframe**	**Spatial setting/ KT form**	**Type of KT activity**	**Social setting/ KT participants**	**Content/ KT processes**	**KT outcomes/outcome use**	**Relevance for DD**
5.	February 6, 2019	Electronic communication (phone call)	User pull	Coordination DD<br><br>Coordination MA	Coordination of additional shadowing sessions in MA	Conceptual:<br>Understanding of how helpful it is to visit the courses on site to better asses how feasible they are at own site	high
					Organization regarding the service agreement	Ø	none
					Organizational arrangements for the ongoing consulting process	Ø	none
					Ideas from coordination MA on the possibility of a zero-cost project extension	Instrumental:<br>Zero-cost project extension to reduce the pressure to adhere to the timeframe and increase project activity feasibility	high
6.	March 13, 2019	Electronic communication (phone call)	User pull	Coordination DD<br><br>Coordination MA	Coordination of further shadowing sessions → sessions in May & July 2019 cancelled due to lack of staff in DD	Conceptual:<br>Arrange consulting sessions outside of shadowing to advance concept development	low
7.	April 8, 2019	Electronic communication (e-mails)	Exchange	Coordination DD<br><br>Coordination MA	Clarification of contractual matters	Ø	none

8.	May 20, 2019<br>2 hours	Electronic communication (phone conference)	User pull	Coordination DD<br><br>Coordination MA<br><br>Physio manager MA	Pre-announced topics via agenda:<br>Project management:<br>Planning meetings, planning kick-off meeting (timing & agenda)<br><br>IT platform for exchange<br><br>Content kick-off meeting<br><br>Discussion on target group kick-off meeting/legal issues: Impact of IP sessions on curricular standards<br><br>Training of IP tutors:<br>Content for presentation<br><br>Networking:<br>Facilitating informal intra-project meetings<br><br>Marketing:<br>Opportunities to publicize IP project, transparency that it is not possible to offer IP sessions for all students during the pilot phase<br><br>Design of IP sessions: Learning objectives and evaluations formulated	Instrumental:<br>Frequency of overall project group meetings & jour fixe working group per course<br><br>Conceptual:<br>Use of a shared drive for all project stakeholders could not be implemented in DD due to IT security regulations<br><br>Instrumental:<br>Kick-off meeting agenda agreed<br><br>Conceptual:<br>Recommendation of agenda kick-off meeting to add call for IP education from medical perspective by doctor with experience of IP work<br><br>Conceptual:<br>Collaboration with teaching department regarding capacity rules and legal issues, consideration of site specifics, coordination with legal department<br><br>Instrumental:<br>Focus on hands-on examples in presen-tation, less about academic issues related to IP sessions<br><br>Conceptual:<br>Informal meeting spaces (e.g. choir, orchestra) in DD not feasible during project period<br><br>Instrumental:<br>Posters, Moodle announcements, announcement in introductory lecture & visits to Carus Academy, creation of project website<br><br>Ø:<br>Recommendation to split up MA learning objectives into subject-specific and	medium<br><br>none<br><br>high<br><br>medium<br><br>high<br><br>high<br><br>none<br><br>medium<br><br>none

Knowledge transfer process							
**Session**	**Timeframe**	**Spatial setting/ KT form**	**Type of KT activity**	**Social setting/ KT participants**	**Content/ KT processes**	**KT outcomes/outcome use**	**Relevance for DD**
						interprofessional learning objectives not used, strong orientation in DD toward draft of National Competence-based Learning Objectives Catalogue for Medicine/ Topic Catalogue for Medicine<br><br>Instrumental:<br>Design evaluation forms in line with development status	 <br><br><br><br>high
9.	June 7, 2019	Material	User pull	Coordination DD<br><br>Coordination MA	MA presentation slides for IP lecture on "Interprofessional patient care"	Conceptual:<br>Internal use of IP lecture working group for stakeholders to help in presentation design	high
10.	August 2019	Electronic communication (telephone calls, e-mails)	User pull	Coordination DD<br><br>Coordination MA	Questions on legally binding text for IP sessions and adaptation of course regulations	Instrumental:<br>Helping with formulations, understanding of which stakeholders at the medical faculty need to be involved	high
11.	January 13, 2020	Bochum: Personal exchange	Exchange	Coordination DD<br><br>Coordination MA	Exchange on the first IP sessions held and potential areas for modification identified<br><br>Exchange on general status, continuation of IP education at faculty	Conceptual:<br>Different assessment of learning gains by professions, minimize heterogeneity from initial knowledge level by choosing a learning objective<br><br>Instrumental:<br>Strategy to create funding for positions to consolidate learning sequences	medium<br><br><br><br><br>high

Knowledge transfer process							
**Session**	**Timeframe**	**Spatial setting/ KT form**	**Type of KT activity**	**Social setting/ KT participants**	**Content/ KT processes**	**KT outcomes/outcome use**	**Relevance for DD**
12.	April 17, 2020<br>2 hours	Electronic communication (video conference)<br><br>Material	User pull	Coordination DD<br><br>Coordination MA<br><br>Head physio departement DD<br><br>Head physio school MA	Pre-announced topics via agenda: Exchange of didactic and methodological challenges in implementing the Clinical Examination Course.<br><br>Possibilities of IP e-learning<br><br>Development of tutor training<br><br>Exchange of experiences at the end of the project	Symbolic:<br>Understanding that IP sessions have to be revised several times and this is ongoing<br><br>Instrumental:<br>Allocation of more time for reflection processes, reduce theoretical parts in IP sessions<br><br>Conceptual:<br>Learning gains in individual sessions can be different between respective professions → pay attention to balance/equalization in overall sequence<br><br>Ø:<br>No e-learning available in MA at time of consultation<br><br>Instrumental:<br>Sending of sample presentation to brief tutors as guidance, note to schedule one-on-one meetings with IP education facilitators to assess needs<br><br>Ø:<br>Recommendation of administrative issues for project completion to be clarified with funding provider	medium<br><br>high<br><br>high<br><br>none<br><br>high<br><br>none
13.	October 12, 2020<br>1 hour	Electronic communication (video conference)	User pull	Coordination DD<br><br>Coordination MA<br><br>*Learning Center manager DD*	Preparation for Study Commission meeting DD: General experiences MA and open question on advice for preparation	Instrumental:<br>Assistance: Focus on content for arguments in favor of IP sessions, overview of previous sessions through project<br><br>Conceptual:<br>Call to form a task force to differentiate discussion of barriers	high<br><br>high

Notes. KT = knowledge transfer, DD = Dresden, MA = Mannheim, Coordination = Coordination of IP education, Physio = physiotherapy, IP = interprofessional, Italics = selectively involved persons.
